# Breast Radiation Recall Phenomenon After Astra-Zeneca COVID-19 Vaccine: A Case Series

**DOI:** 10.7759/cureus.21499

**Published:** 2022-01-22

**Authors:** Rory Marples, Cameron Douglas, Joseph Xavier, Allen-John Collins

**Affiliations:** 1 Surgery, South East Regional Hospital, Bega, AUS; 2 General Surgery, South East Regional Hospital, Bega, AUS

**Keywords:** breast cancer, medical oncology, radiation oncology, breast surgery, surgery, general surgery

## Abstract

We report three cases of AstraZeneca vaccine (AZV)-induced radiation recall phenomenon (RRP) in three women who had previously undergone radiotherapy for breast cancer. RRP is a rare complication of vaccination that can mimic the more common pathology of breast cellulitis. Emergency physicians, primary care specialists, and surgeons should be aware of RRP when treating patients in the current coronavirus disease 2019 (COVID-19) climate.

## Introduction

In the Breast and Endocrine Surgery department at South East Regional Hospital, New South Wales, we observed three women with the diagnosis of grade one-two radiation recall phenomenon (RRP) after receiving a single dose of the AstraZeneca vaccine (AZV). All three women had undergone breast surgery and radiation therapy for cancer in the preceding year, and all were in remission at the time of writing. RRP is a rare, self-limiting pathology whereby breast tissue undergoes a latent reactivation, causing a painful dermatitic rash in the field of previously irradiated tissue, regardless of the arm-injection site for the vaccine. The cases were significant due to all being initially confused with the more commonly seen cellulitis of the breast, which was ruled out on further clinical examination and investigation, leading to a diagnosis of RRP. This is an important first step in understanding vaccine side-effects for patients with significant comorbidities such as breast cancer, and the risk profile they carry for future vaccines.

## Case presentation

Case one

**Figure 1:**
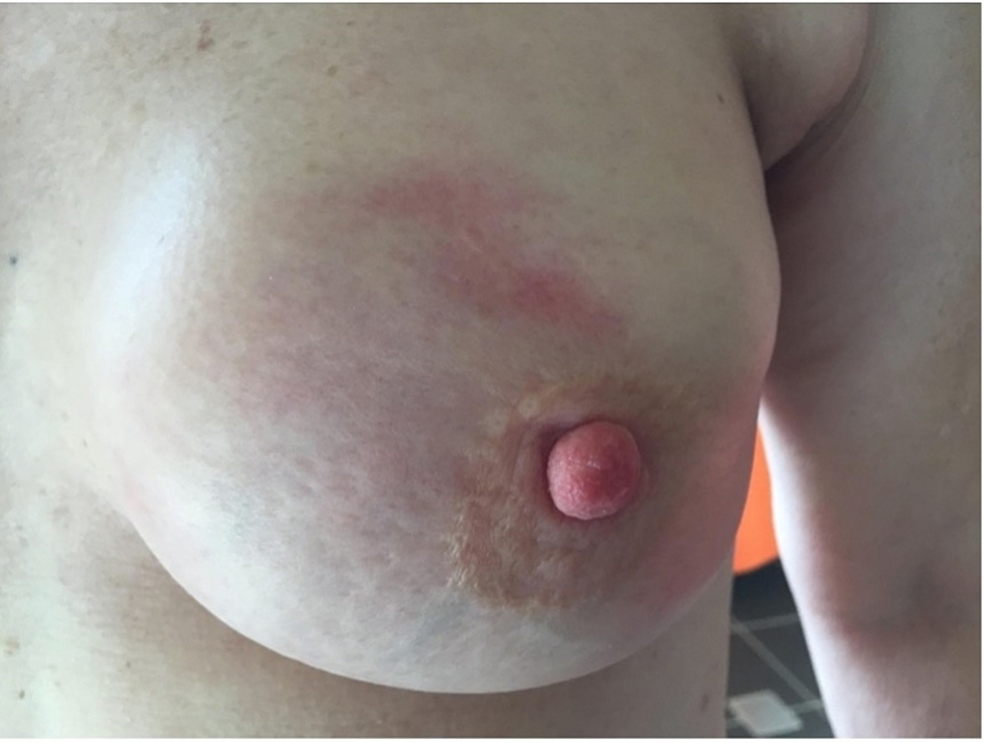
Acute skin reaction (Patient 1) three days after the vaccine

**Figure 2:**
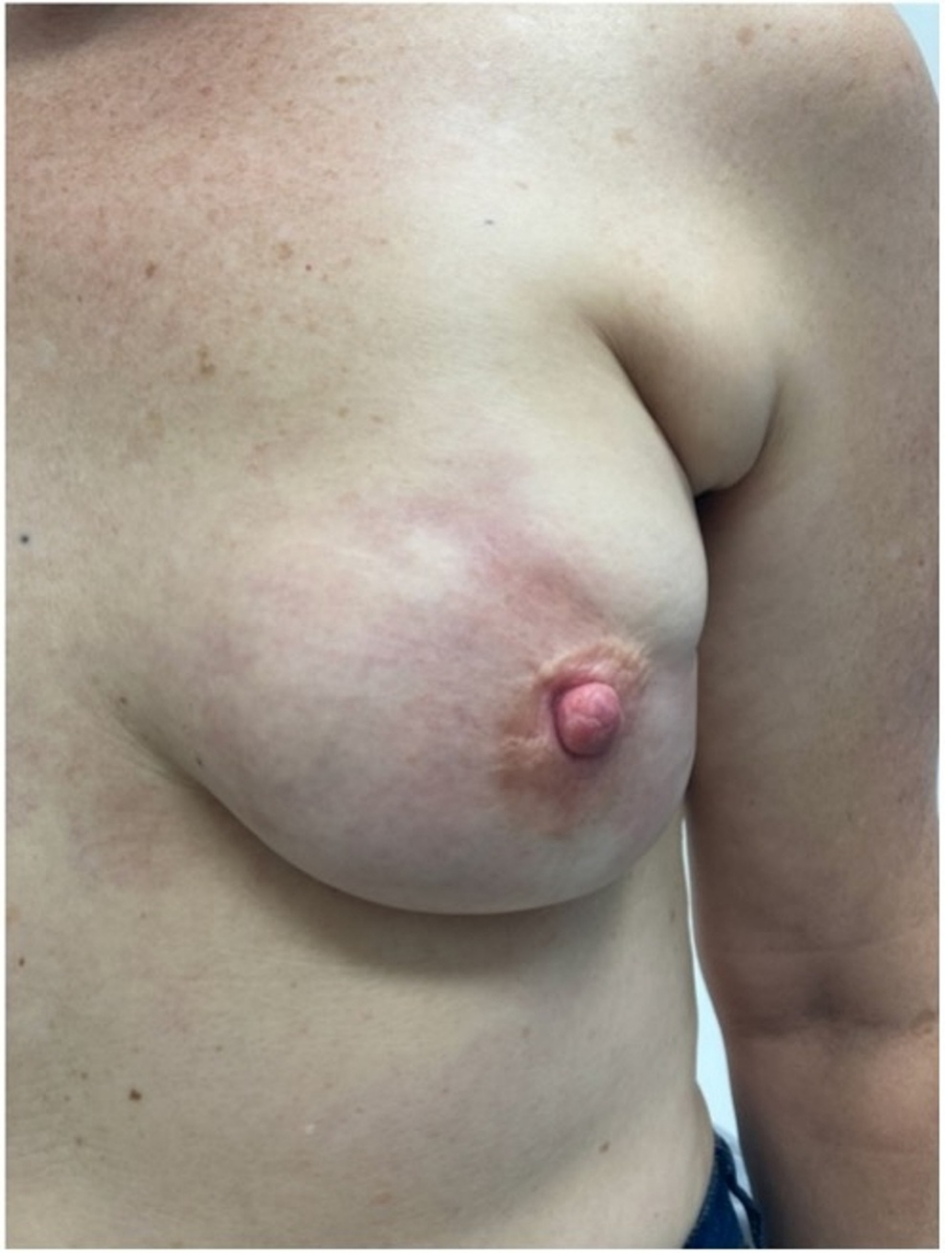
Patient 1 three months after the first AZ vaccine, two weeks before the second dose, with prednisone treatment already commenced

Case one is a 62-year-old woman with a background of left breast cancer, who underwent a bilateral mastectomy with reconstruction, left axillary clearance, and adjuvant radiotherapy in 2018. In May of 2021, the patient had her first dose of the coronavirus disease 2019 (COVID-19) AZV. This was administered to her right arm due to contralateral lymphoedema. Three days later, she presented to the outpatient surgical clinic with swelling, erythema, and pain in her left breast (Figure 1). She did not have a fever and was not systemically unwell. She was later reviewed by her medical oncologist and commenced on 50 mg prednisone daily, tapered to 12.5 mg daily over one week, and then increased again to 25 mg daily due to ongoing erythema. Her total course of corticosteroid was four weeks. At a follow-up appointment four weeks later, the rash had improved considerably (Figure 2), and the patient no longer reported pain in the area. After review by her surgeon and the medical oncologist, the patient was cleared to receive her second dose of the AZV and no further symptoms were reported.

Case two

**Figure 3:**
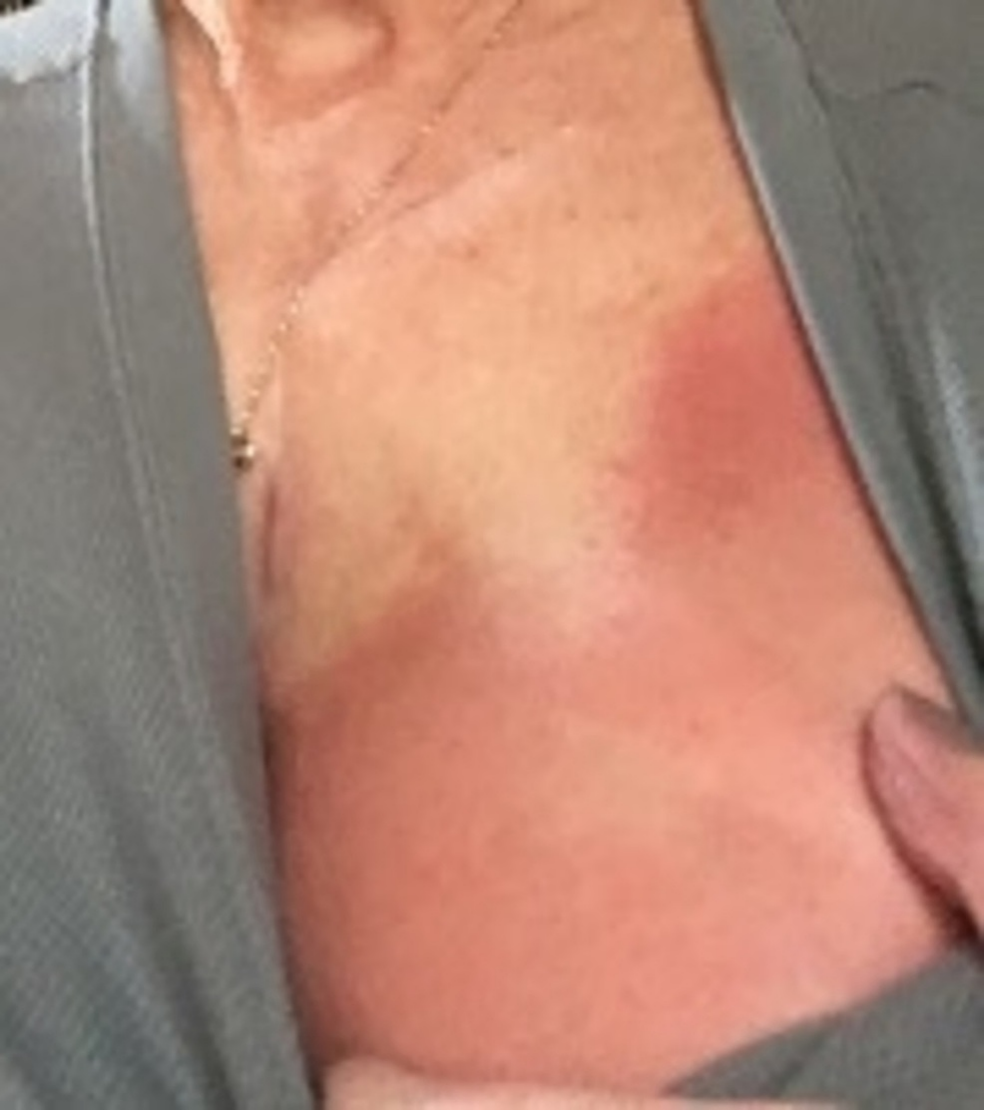
Acute dermatitic reaction overlying the left breast (Patient 2) two weeks following the first dose of the AZV

**Figure 4:**
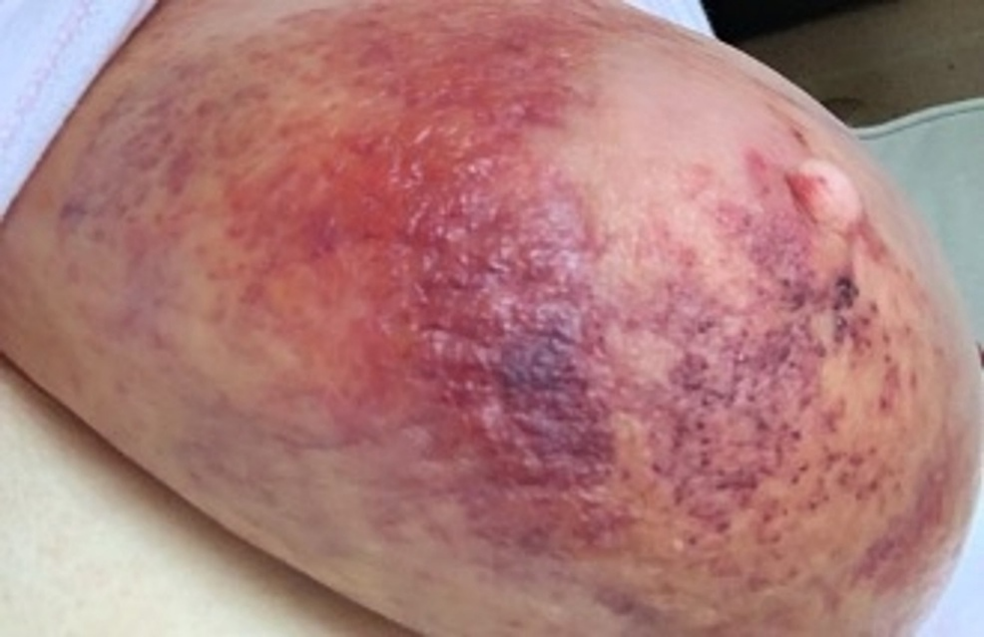
Second episode of RRP in the same patient (case two) RRP: radiation recall phenomenon

A 69-year-old woman received adjuvant radiotherapy for left breast cancer following lumpectomy and sentinel node biopsy. The patient received the AZV in April of 2021. Three days later, she developed systemic symptoms, including fever, muscle aches, and lethargy. However, over the subsequent two days, she experienced pain and erythema in the left breast, with bilateral axillary pain. On examination, her vital signs were all within normal limits and she was afebrile. She had erythema covering most of her left breast, with mild induration and small blood blisters superiorly. The breast was tender, however, there was no fluctuance to suggest an underlying collection (Figure 3). She also had palpable, tender lymph nodes in the bilateral axillae. Given her systemic symptoms and history of a mitral valve replacement (for which she takes warfarin), the patient was admitted to the hospital for observation and a breast ultrasound was ordered to rule out an underlying collection. Following a negative ultrasound, normal full blood count and biochemistry, and the close temporal relationship between her vaccine dose and the rash, the patient was given a provisional diagnosis of radiation recall phenomenon and discharged from the hospital with simple analgesia and follow-up in the general surgery clinic. The patient then reported two further episodes of erythema and swelling in her left breast, five and seven weeks after her first AZV dose, respectively (Figure 4). She presented for follow-up in the general surgery clinic in June of 2021 with no evidence of RRP to her left breast.

Case three

**Figure 5:**
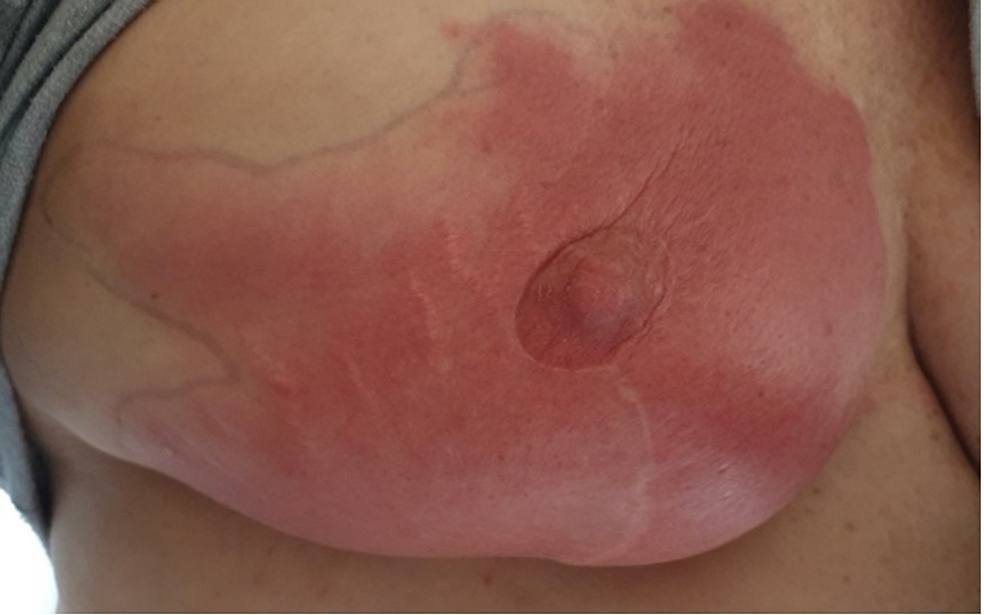
Three days after COVID-19 vaccination with AZV

**Figure 6:**
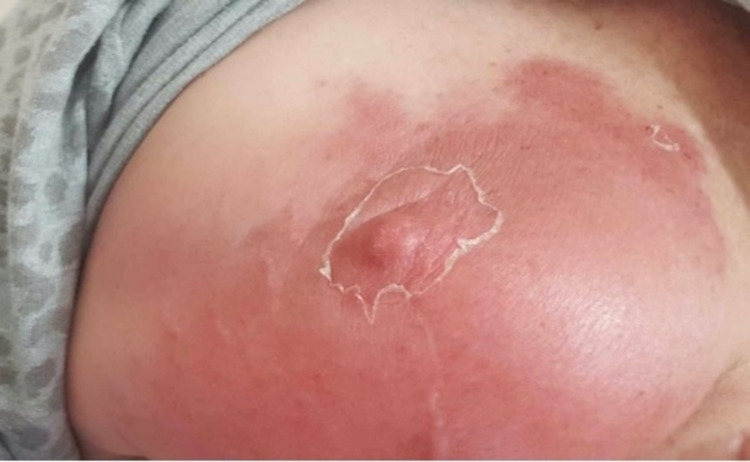
Four days after vaccination, with notable blistering around the nipple-areolar complex

**Figure 7:**
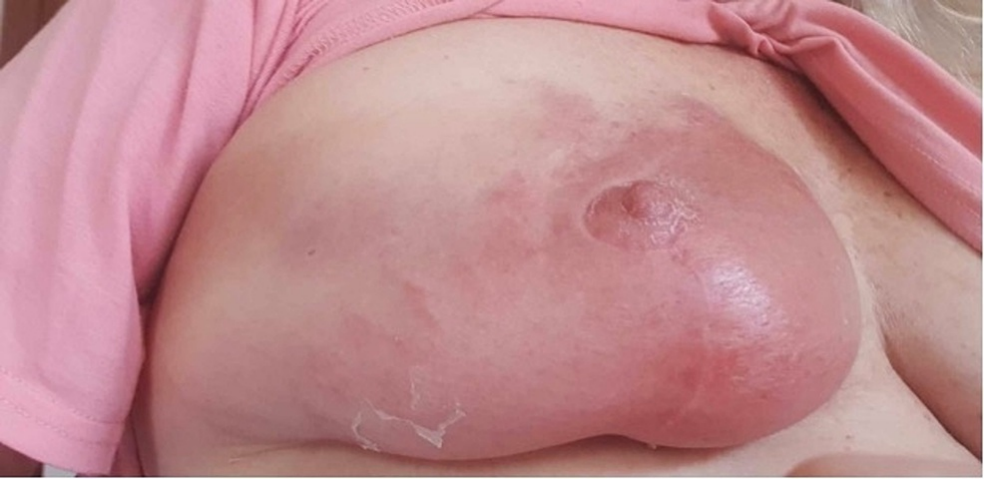
Seven days after vaccination. There is a small amount of resolution to the rash, with ongoing blistering to the right lower quadrant

Our third case is a 56-year-old lady who had previously undergone a therapeutic reduction mammoplasty and sentinel node biopsy for a multifocal lobular carcinoma of the right breast in April 2021. This was followed by whole breast radiotherapy, following which she recovered well. Three months later, the patient received the first dose of the AZV, and, soon after, developed flu-like symptoms. Three days later, she reported a hot, itchy, and heavy right breast in a similar area where she had previously received radiation therapy (Figure 5). She was commenced on augmentin Duoforte 1 tablet twice daily by her GP for the rash and subsequently spent two days recuperating at home with improvements in her symptoms. An ultrasound was performed at the time to exclude underlying collection, which demonstrated subcutaneous edema consistent with cellulitis and no drainable collection. She was reviewed by her breast surgeon one week after her vaccine was administered, and a diagnosis of radiation recall phenomenon was made (Figures 6-7).

## Discussion

RRP is a rare complication seen in previous therapeutically irradiated tissue soon after the administration of a pharmacological agent [1]. The first case of RRP was described by D’Angio et al. in 1959 at the Children’s Medical Centre in Boston, USA [2], where he and his colleagues described the latent potentiation of previous X-ray effects on normal tissues after administering a dose of actinomycin D. It involves a latent sub-acute inflammatory of the irradiated area, most commonly a dermatitic reaction in the skin, although RRP in the lung, small intestine, and muscle have also been reported [1]. Since D’Angio’s finding, many other cytotoxic drugs have been associated with RRP, including paclitaxel, methotrexate, 5-fluorouracil, and bleomycin [3], however, it can occur with almost any medication [1]. Despite RRP being well-documented in the literature, little is known about its mechanism.

Four distinct aetiologies have been proposed that may account for RRP, although there is little evidence to support these theories: 1) vascular damage, 2) epithelial stem cell inadequacy, 3) epithelial stem cell sensitivity, and 4) drug hypersensitivity reaction [3]. Camidge et al. have subsequently proposed a grading system for RRP based on the Radiation Therapy Oncology Group (RTOG) Acute Radiation Morbidity Criteria, with common RRP systems, added: Grade 1: Erythema +/- pruritis +/- dry desquamation, grade 2: grade 1 with pain, edema, urticaria, or desquamation, grade 3: moist desquamation, grade 4: Necrosis, ulceration, or hemorrhage.

Vaccine-related RRP remains a rarer and even less understood phenomenon. Soyfer et al. have reported a world first of RRP in two patients who received the mRNA Pfizer COVID-19 vaccination [4]. In their case series, two patients who had received the Pfizer vaccine presented to their clinic with a painful, erythematous rash in the distribution of the radiation field, prompting a diagnosis of RRP.

Since collating our cases, Stewart et al. described a patient who received the first of two doses of the AZV six months after completing radiation therapy for acinic cell carcinoma of the right parotid gland. Three hours after the injection, the patient had the onset of a pruritic, dermatitic rash over her right upper neck in the radiation field, which persisted for three weeks with subsequent desquamation requiring dressings and oral corticosteroid therapy [5]. Our cases are similar in the use of corticosteroid therapy, however, both indicate how RRP is a self-limiting condition that will improve over time. At the time of writing, all COVID-19 vaccines appear to be associated with some form of RRP, indicating the ubiquity of the condition and how important it is for clinicians to be aware of this complication [6].

## Conclusions

We describe three patients with grade one-two RRP after receiving their first dose of the AZV, in a background of breast cancer treated with radiotherapy. This case report is limited by the small sample size of three patients. However, it is an important first step in understanding potential side-effects of the AZV, particularly in patients with a history of radiotherapy and intercurrent medical conditions. Further understanding is required to properly identify this phenomenon and provide useful information to patients, particularly as they consider the second and booster doses of a vaccine.
